# Crop Diversification for Ensuring Sustainable Agriculture, Risk Management and Food Security

**DOI:** 10.1002/gch2.202400267

**Published:** 2025-01-12

**Authors:** Tesfahun Belay Mihrete, Fasikaw Belay Mihretu

**Affiliations:** ^1^ Department of Horticulture College of Agriculture and Environmental Sciences Bahir Dar University Bahir Dar 6000 Ethiopia

**Keywords:** agricultural risk, climate resilience, food security, market volatility, soil health

## Abstract

Agriculture faces growing challenges from climate change, pest pressures, and market instability. Crop diversification offers a sustainable strategy to enhance resilience and reduce the risks of monoculture. This review examines crop diversification as a response to these challenges, with a focus on its applications in sustainable agriculture, risk management, and food security. Strategies such as spatial, temporal, genetic, and intercropping diversification enhance soil health, improve pest management, and boost resilience to climate variability. The review highlights key principles, including ecological resilience, risk distribution, and resource optimization. By adopting diverse crops, farmers can mitigate soil degradation, reduce pest outbreaks, and stabilize incomes. Successful case studies from various regions, such as integrated rice‐fish farming and agroforestry, demonstrate how diversification can improve productivity and sustainability. However, challenges remain, such as knowledge gaps, market access issues, and policy limitations. The review concludes with recommendations for future research and policy interventions, stressing the need for tailored diversification strategies, better support systems, and further exploration of innovative practices. This overview underscores the potential of crop diversification to build resilient, sustainable agricultural systems while addressing global food security concerns.

## Graphical abstract

Crop diversification offers a sustainable pathway to address agriculture's pressing challenges, such as climate change, pest pressures, and market volatility. This review explores its role in promoting soil health, managing risks, and ensuring food security through spatial, temporal, and genetic diversification strategies, supported by successful case studies like agroforestry and rice‐fish farming.

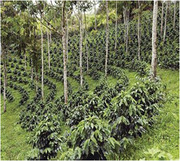

## 1 Introduction

Agriculture, a foundational pillar of human civilization, faces unprecedented challenges in the 21^st^ century.^[^ ^1^ ^]^ The accelerating impacts of climate change, rising pest and disease pressures, and unstable market conditions demand significant adaptations to secure food supplies, ensure environmental sustainability, and maintain economic stability (**Table** **1**). Climate change poses a primary threat to global agriculture.^[^ ^2^ ^]^ Shifting weather patterns, rising temperatures, and the increased frequency of extreme weather events such as droughts and floods disrupt traditional farming methods.^[^ ^2^ ^]^ These changes can negatively influence crop yields, alter growing seasons, and increase susceptibility to pests and diseases. Higher temperatures can stress crops sensitive to heat, while unpredictable rainfall can complicate irrigation and affect soil moisture (Table 1). Furthermore, intensified agriculture and global trade expansion have facilitated the spread of pests and diseases, posing additional risks to crops.^[^ ^3^ ^]^ Monoculture systems provide ideal conditions for pests and diseases to proliferate, leading to rapid spread and significant losses.^[^ ^4^ ^]^ Overreliance on chemical controls can also lead to resistance among pests and pathogens due to repeated exposure.^[^ ^5^ ^]^ Market volatility, driven by seasonal supply changes, global trade policies, and shifting consumer preferences, further exacerbates these challenges.^[^ ^6^, ^7^, ^8^ ^]^ Price fluctuations can severely influence farmers’ incomes, particularly those dependent on single crops, leading to financial instability and jeopardizing the viability of agricultural enterprises.^[^ ^8^ ^]^

**Table 1:** Challenges to Agriculture and the Mitigating Role of Crop Diversification.

Challenge	Supporting Data	Impact	Mitigating Role of Crop Diversification
Soil Depletion	Global Soil Degradation: Over 33% of the world's soils are degraded, affecting 1.5 billion people.^[^ ^15^ ^]^	Reduced soil fertility and water retention lead to lower productivity and higher risk of crop failure.	Diversification helps maintain soil health by improving nutrient cycling and reducing erosion.
	Nutrient Loss: Monoculture can reduce crop yields by up to 20% due to nutrient depletion.^[^ ^16^ ^]^		
Disease and Insect Pest and Pressure	Economic Impact: Crop pests and diseases cause $220 billion in annual losses.^[^ ^17^ ^]^	Increased crop losses and severe pest outbreaks affect yields and food availability.	Crop diversification reduces pest pressure and resistance by disrupting pest habitats and life cycles.
	Increased Pest Resistance: Monocultures can heighten pest problems and foster resistance to chemical controls by repeatedly exposing pests to the same chemicals.^[^ ^18^ ^]^		
Food Insecurity	Global Food Insecurity: Nearly 828 million people are undernourished, and 2.3 billion suffer from food insecurity.^[^ ^19^ ^]^	Poor nutrition and stunted growth from mono‐diets reduce productivity and economic potential, perpetuating poverty and affecting future generations' health.	Diversification improves food security by increasing production stability and dietary variety.
	Impact of Monoculture: Reliance on single crops can lead to food system vulnerabilities.^[^ ^20^ ^]^		
Climate Change	Climate Effects: Global temperatures have risen by approximately 1.1 °C since the pre‐industrial era.^[^ ^21^ ^]^	Altered growing conditions and increased frequency of extreme weather events threaten crop yields.	Diversification can increase resilience to climate variability by spreading risk across different crop types suited to varying conditions.
Market Price Volatility	Price Fluctuations: Agricultural commodity prices can fluctuate by over 30% annually.^[^ ^22^ ^]^	Unstable prices lead to income uncertainty for farmers and can influence food affordability for consumers.	Diversification reduces economic risk by spreading income sources and reducing dependence on a single crop's market performance.

Compounding these issues is the persistent problem of food insecurity, especially prevalent in developing regions such as Sub‐Saharan Africa and South Asia, where malnutrition and stunting in children remain critical concerns.^[^ ^9^ ^]^ Monoculture farming—cultivating a single crop over extensive areas—contributes significantly to these challenges.^[^ ^9^ ^]^ It often results in degraded soil health, increased vulnerability to pests and diseases, and reduced resilience to climate variability.^[^ ^9^, ^10^ ^]^ This practice depletes soil nutrients and makes crops more susceptible to environmental stress. Additionally, limited access to modern farming techniques further exacerbates these issues, perpetuating food insecurity in these regions.^[^ ^10^ ^]^

**Figure 1:**
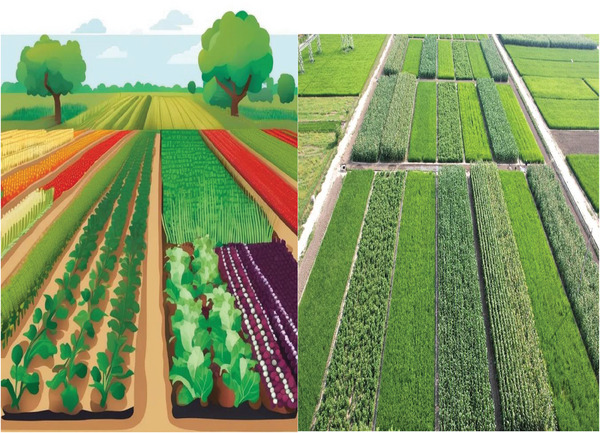
Crop Diversification in Agriculture as Manifested by Inter‐cropping and Agro‐forestry Practices. Reproduction with permission from Singh et al.**^[^** ^12^ ^]^

To tackle these challenges, crop diversification has emerged as a viable strategy.^[^ ^11^ ^]^ This approach involves growing a variety of crops within the same farm or across different regions, rather than relying on a single crop as manifested by Intercropping (strip, row, mixed, alley and relay), Agroforestry practices (Alley Cropping, Silvopasture, Riparian Forest Buffers, Windbreaks and forest farming) (**Figure** **1**).^[^ ^12^ ^]^ Strategies such as spatial diversification (planting different crops in separate fields), temporal diversification (rotating crops through different seasons or years), genetic diversification (using various crop varieties), and intercropping (growing multiple crops together in the same field) enable farmers to spread risk and mitigate adverse impacts.^[^ ^13^ ^]^ Diverse cropping systems enhance soil health, improve pest management, boost biodiversity, and reduce dependence on chemical inputs. These benefits align with broader goals of sustainable agriculture, including environmental health, economic stability, and food security.^[^ ^14^ ^]^ As global interest in high‐value crops grows, driven by concerns over biodiversity loss and environmental health, there is a need for comprehensive information on how crop diversification can support agricultural sustainability. This review aims therefore to provide an in‐depth overview of crop diversification efforts, exploring its role in agricultural sustainability, risk management, and food security, while highlighting its benefits, challenges, and future directions.

## 2 Key Principles and Approaches Guiding Effective Crop Diversification

Crop diversification is guided by several core principles that underscore its role in promoting sustainable agriculture. One fundamental principle is ecological resilience, which emphasizes the importance of biodiversity in maintaining stable and functional ecosystems.^[^ ^23^ ^]^ By incorporating a variety of plant species, farmers can enhance their system's ability to withstand and recover from environmental stresses such as pests, diseases, and climate variability.^[^ ^24^ ^]^ For instance, diverse cropping systems often support a range of organisms, including natural pest predators, which help control pest populations and reduce the need for chemical pesticides.^[^ ^24^ ^]^ This approach fosters a balanced ecosystem where multiple plant species disrupt pest life cycles and mitigate the risk of large‐scale infestations.^[^ ^23^ ^]^ Another crucial principle is risk management. This involves distributing economic risk by cultivating a variety of crops rather than relying on a single crop.^[^ ^25^ ^]^ This diversification strategy helps minimize potential financial losses due to the failure of any one crop. The principle operates on the notion that while one crop may underperform, others may thrive or at least offset losses, thus promoting overall financial stability.^[^ ^25^ ^]^ Research supports that diversified farming systems provide greater economic resilience. For example, farmers who grow a mix of cereals, legumes, and vegetables are better equipped to handle adverse conditions or market fluctuations, thereby reducing their vulnerability to economic shocks.^[^ ^26^ ^]^

Resource optimization is another central principle of crop diversification. This principle focuses on the efficient use of resources such as soil nutrients, water, and land.^[^ ^27^ ^]^ By leveraging the varying nutrient requirements and growth patterns of different crops, farmers can maintain a balanced nutrient profile in the soil and minimize competition for resources.^[^ ^28^ ^]^ Key practices under this principle include crop rotation and intercropping.^[^ ^28^ ^]^ For instance, rotating nitrogen‐fixing legumes with cereals improves soil fertility by adding nitrogen and lessening the need for synthetic fertilizers. Similarly, intercropping—where farmers plant crops with different resource needs together—can enhance soil health and water use efficiency.^[^ ^29^ ^]^ Productivity and yield stability are also vital aspects of crop diversification.^[^ ^30^ ^]^ Diversified cropping systems tend to be more resilient to environmental stresses and can lead to higher consistent yields over time than monoculture systems.^[^ ^31^ ^]^ This principle is particularly pertinent in the context of climatic variability.^[^ ^31^ ^]^ For example, intercropping multiple crops can buffer against environmental stresses such as droughts or floods. By utilizing crops with diverse growth patterns and resource needs, these systems can reduce yield fluctuations and contribute to more stable agricultural outputs.^[^ ^32^ ^]^

Farmers commonly use several approaches to implement crop diversification effectively.^[^ ^33^ ^]^ Species diversification involves growing different species of crops together within the same area, enhancing pest control and resource utilization.^[^ ^33^ ^]^ The traditional “Three Sisters” planting technique, which combines maize, beans, and squash, exemplifies this approach. Maize supports beans, beans fix nitrogen in the soil, and squash covers the ground to suppress weeds and retain moisture.^[^ ^34^ ^]^ Another approach is varietal diversification, which involves using multiple varieties of the same crop to enhance genetic diversity and resilience. This method helps manage specific pests and diseases that may affect only certain varieties.^[^ ^35^ ^]^ For example, planting different wheat varieties with varying disease resistance can reduce the risk of widespread crop failure and enhance system resilience.^[^ ^35^ ^]^ Spatial diversification includes practices such as intercropping and agroforestry.^[^ ^36^ ^]^ Intercropping involves planting different crops close together to optimize land use and resource efficiency.^[^ ^37^ ^]^ For instance, intercropping legumes with cereals can boost soil fertility and reduce pest pressure.^[^ ^37^ ^]^ Agroforestry integrates trees with crops, offering additional benefits like improved soil structure and supplementary income from timber or fruit.^[^ ^38^ ^]^ Temporal diversification approach is also used which focuses on varying the timing of planting and harvesting to extend the growing season and maximize resource use.^[^ ^39^ ^]^ Sequential cropping, where farmers grow different crops in succession on the same land, allows for more efficient land use and continuous crop production.^[^ ^40^ ^]^ Staggering planting times helps manage risks and ensures a steady supply of produce throughout the year.^[^ ^41^ ^]^ Finally, landscape‐level diversification involves implementing various cropping systems across different fields or plots within a larger landscape.^[^ ^42^ ^]^ This broader approach can enhance ecological balance and optimize resource use across the entire farm.^[^ ^42^ ^]^ For example, integrating different cropping practices—such as annual crops, perennials, and cover crops—across various farm areas can improve biodiversity and overall farm sustainability.^[^ ^42^, ^43^ ^]^

## 3 Advantages of Crop Diversification: Promoting Sustainable Agriculture Through Enhanced Soil Health, Pest Management, and Economic Resilience

Crop diversification, which involves growing a range of different crops within a single farming system or across multiple growing seasons, is increasingly recognized as a fundamental approach to sustainable agriculture.^[^ ^44^ ^]^ This practice offers a range of benefits, from boosting soil health to improving economic stability and managing pests. A key benefit of crop diversification is its significant impact on soil health. Incorporating a variety of crops, such as legumes, can greatly enhance soil fertility.^[^ ^45^ ^]^ The mechanism behind this enhancement lies in the ability of legumes to fix atmospheric nitrogen through a symbiotic relationship with Rhizobium bacteria in their root nodules. This process converts inert nitrogen into a bioavailable form that enriches the soil, reducing the need for synthetic fertilizers.^[^ ^46^ ^]^ Furthermore, the natural nitrogen fixation by legumes is crucial for maintaining soil vitality, as it replenishes the soil with nitrogen after crops that deplete it.^[^ ^47^ ^]^

**Figure 2:**
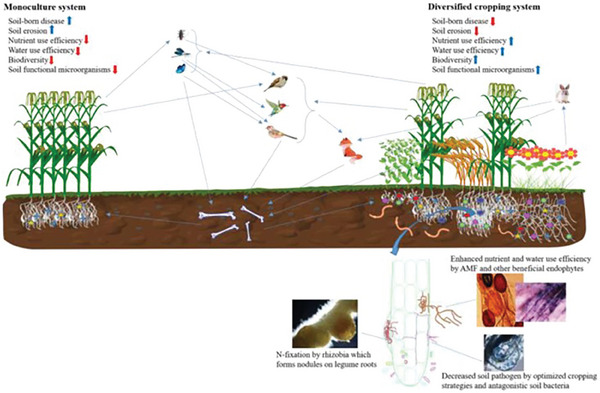
Soil health comparison in diversified cropping systems and monocultures. Reproduction with permission from Aulakh and Malhi.^[^ ^49^ ^]^

In addition to enhancing nitrogen levels, the diverse root structures of different crops, which vary in depth and architecture, create a more complex soil environment. For instance, deep‐rooted crops can access nutrients from deeper soil layers, while shallow‐rooted crops utilize surface nutrients.^[^ ^48^ ^]^ These varying root systems help to prevent soil erosion, as deeper roots anchor the soil, while the surface roots of other crops reduce water runoff.^[^ ^48^ ^]^ This variety also boosts organic matter content in the soil, as the decomposition of roots from different crops contributes to soil structure and fertility. Moreover, the reduced nutrient competition among crops with different nutrient requirements supports more efficient nutrient use, preventing depletion of soil resources (**Figure** **2**).^[^ ^49^ ^]^ This complementary interaction between crops contributes to overall soil health, making it more resilient and productive over time. Furthermore, studies by Yin et al.^[^ ^50^ ^]^ and Mrabet et al.^[^ ^51^ ^]^ underscore that a well‐planned crop rotation—which leverages the complementary growth habits and nutrient demands of different crops—can enhance soil structure, promote microbial diversity, and reduce pest buildup, leading to healthier soils and more sustainable farming practices.

In the realm of pest and disease management, the strategy of crop diversification is highly effective in disrupting the cycles of pests and diseases.^[^ ^52^ ^]^ The mechanism behind this effect is rooted in the creation of a more complex, heterogeneous farming environment. By varying crop types within a system, this approach reduces the prevalence of pests and diseases by breaking the continuity of host crops that pests and pathogens depend on.^[^ ^53^ ^]^ For example, when crops are rotated or intercropped, pests that specialize in one type of plant are less likely to find a continuous food source.^[^ ^52^ ^]^ This interruption in the pest lifecycle prevents rapid population buildup, making pest management more sustainable. Additionally, the presence of a variety of crops offers habitats for natural predators such as ladybugs, predatory beetles, and parasitoid wasps, which help control pest populations by preying on the pests or their larvae. This biological control is crucial in reducing the need for chemical pesticides.^[^ ^54^ ^]^ He et al.^[^ ^55^ ^]^ highlighted that enhancing crop diversity significantly diminishes pest outbreaks, thereby reducing the dependence on chemical controls, which is beneficial for both the environment and farm economics. Similarly, Huss et al.^[^ ^56^ ^]^ and Toker et al.^[^ ^57^ ^]^ illustrated that intercropping—the practice of growing different crops together—fosters ecological interactions that reduce pest pressures and curb disease spread. By planting crops with different susceptibility profiles, the risk of pests and diseases spreading across the entire farm is reduced.^[^ ^58^ ^]^ Certain plants may also produce volatile organic compounds that deter pests or attract pest predators, further disrupting pest lifecycles and reducing the need for chemical intervention. This creates a more robust agricultural environment, as the ecological diversity within the system helps maintain natural balance and promotes resilience to pest and disease outbreaks.^[^ ^59^ ^]^ Another significant advantage of crop diversification is its positive impact on resource efficiency. Different crops have distinct needs for water, light, and nutrients, and cultivating them together can lead to a more efficient use of these resources.^[^ ^60^ ^]^ The mechanism behind this efficiency lies in the complementary growth patterns and resource requirements of the intercropped species. For instance, deeper‐rooted crops can access nutrients from lower soil layers, while shallow‐rooted crops use nutrients from the topsoil.^[^ ^61^ ^]^ This resource partitioning minimizes competition and maximizes the overall resource use across the system. Moreira et al.;^[^ ^62^ ^]^ Leonel et al.;^[^ ^63^ ^]^ Kuyah et al.^[^ ^64^ ^]^ note that intercropping improves water and nutrient utilization, as crops can exploit different niches within the same space, leading to reduced waste and more efficient management of agricultural inputs. This resource‐use complementarity is essential for conserving water and nutrients, which are critical inputs in sustainable agricultural practices.^[^ ^65^ ^]^ Moreover, the reduced dependency on external inputs like fertilizers and water due to more efficient use of available resources contributes to the sustainability of agricultural systems.^[^ ^32^ ^]^

Additionally, crop diversification boosts biodiversity and resilience within agricultural ecosystems. According to Smith et al.^[^ ^66^ ^]^ varied cropping systems support greater biodiversity and ecosystem functionality compared to monocultures. The mechanism behind this is that diverse cropping systems provide a range of habitats and resources that support a wider variety of species. By nurturing a wide array of life forms—such as soil microorganisms, birds, amphibians, reptiles, and small mammals—diverse systems create a more complex and interconnected ecosystem.^[^ ^67^ ^]^ This complexity enhances ecological stability by supporting a variety of functional roles within the system, from nutrient cycling to pest control.^[^ ^68^ ^]^ Soil microorganisms, including bacteria and fungi, play a pivotal role in nutrient cycling and soil health, which directly influences soil fertility and structure.^[^ ^69^ ^]^ The diversity of these microorganisms within a more varied cropping system supports more efficient breakdown of organic matter, enhances nutrient availability, and promotes soil structure, which is vital for healthy crop growth.^[^ ^70^ ^]^ By contrast, monocultures tend to favor a narrow set of microorganisms, potentially reducing the efficiency of nutrient cycling and making the soil more vulnerable to pests and diseases.^[^ ^71^ ^]^

Birds and small mammals also help manage pest populations and maintain ecosystem equilibrium by consuming pests and acting as natural regulators of food webs.^[^ ^72^ ^]^ These animals benefit from the variety of food sources and habitats offered by diversified farming systems.^[^ ^72^ ^]^ The presence of such animals contributes to natural pest control, reducing the need for chemical pesticides and promoting ecological balance. Amphibians and reptiles benefit from reduced pesticide use, which supports their habitats and fosters a more stable ecosystem.^[^ ^73^ ^]^ These organisms are sensitive to pesticide exposure, and crop diversification reduces the overall pesticide load, thereby protecting their habitats and ensuring that their populations thrive. The preservation of amphibians and reptiles in turn benefits the ecosystem by maintaining biodiversity and regulating pest species.^[^ ^74^ ^]^ Collectively, these diverse organisms contribute to a resilient and adaptable agricultural environment by increasing biodiversity, fostering interactions between species, and ensuring that ecosystem functions are maintained even in the face of environmental changes.^[^ ^75^ ^]^ Furthermore, crop diversification provides substantial advantages for pollinator support. A diverse range of flowering plants attracts a variety of pollinators, such as bees, butterflies, and other insects, which are essential for pollination services across different crops.^[^ ^76^ ^]^ The variety of plants and flowers in a diversified system ensures that pollinators have access to a constant and varied food source, which supports their populations year‐round.^[^ ^77^ ^]^ Garibaldi et al.^[^ ^78^ ^]^ and Bartomeus et al.^[^ ^79^ ^]^ demonstrated that greater floral diversity in agricultural settings leads to higher pollinator populations and improved crop yields, underscoring the direct benefits of incorporating a variety of flowering plants into agricultural practices.

**Figure 3:**
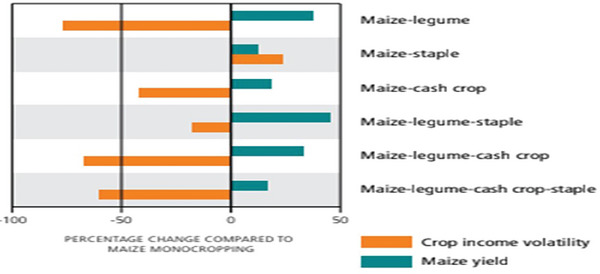
Diversified systems, particularly those that incorporate legumes, significantly reduce crop income volatility compared with maize mono cropping. Reproduction with permission from Mortensen and Smith.^[^ ^81^ ^]^

From an economic perspective, crop diversification enhances stability by mitigating risks related.^[^ ^80^ ^]^ As depicted in **Figure** **3**, farms with diverse cropping systems are less susceptible to economic volatility compared to monoculture systems.^[^ ^81^ ^]^ This is because diverse systems can spread risk across different crops, reducing the impact of price fluctuations or pest outbreaks on the entire farm.^[^ ^81^ ^]^ Tadele^[^ ^82^ ^];^ Birthal and Hazrana^[^ ^83^ ^]^ found that diversified farms tend to have more stable income streams, even in times of market or environmental shocks. Polzin et al.^[^ ^84^ ^]^ and Hallegatte^[^ ^85^ ^]^ show that these diversified systems offer more stable income, reducing financial risks and contributing to long‐term economic resilience. The ability to grow multiple crops also means farmers can adapt to changing market demands, thus ensuring that income remains steady even in the face of challenges.^[^ ^85^ ^]^

Implementing crop diversification in practice involves a range of strategic approaches. A crucial first step is the thoughtful selection of crops with complementary growth characteristics and resource requirements.^[^ ^86^ ^]^ The mechanism behind this is that selecting crops with complementary traits—such as varying root depths, nutrient requirements, and growth habits—allows the crops to utilize different ecological niches within the same field. For example, some crops may have deep root systems that access nutrients deeper in the soil, while others may have shallow roots that utilize nutrients closer to the surface. This minimizes resource competition and promotes optimal resource allocation, enhancing overall system productivity and reducing the risk of soil degradation. Incorporating both annual and perennial plants, along with cover crops, can further bolster soil health and system resilience.^[^ ^87^ ^]^ Perennials have deeper root systems that can help prevent soil erosion and maintain soil structure over time, while annuals can provide quick returns and a diverse range of ecological functions.^[^ ^87^ ^]^ Cover crops, which are often planted during off‐seasons, contribute to soil fertility by adding organic matter, preventing soil erosion, and reducing weed pressure. Together, these practices create a more resilient and sustainable farming system, improving soil health and reducing the need for synthetic inputs.^[^ ^88^ ^]^ In addition to this, methods such as crop rotation and intercropping are instrumental in managing soil health and boosting productivity.**^[^** ^89^ ^]^ Crop rotation involves changing the types of crops grown on a particular piece of land each season or year, which disrupts pest and disease cycles and helps prevent the depletion of specific soil nutrients. The mechanism here is that rotating crops with different nutrient requirements (e.g., legumes that fix nitrogen versus cereals that require large amounts of nitrogen) allows the soil to naturally replenish and avoid nutrient imbalances. This practice also helps reduce soil‐borne diseases, as pests and pathogens specific to certain crops are less likely to build up in the soil when their host crops are rotated out.^[^ ^90^ ^]^

**Figure 4:**
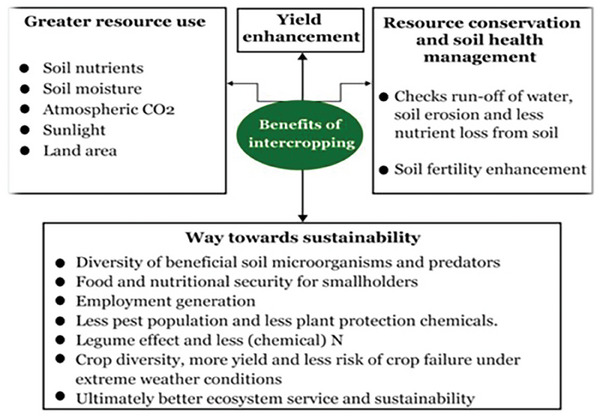
Benefits of Intercropping in Enhancing Resource Use Efficiency, Promoting Resource Conservation, and Improving Soil Health, All Contributing to Sustainable Agricultural Practices. Reproduction with permission from Stomph et al.^[^ ^91^ ^]^

Meanwhile, intercropping, or growing two or more crops together in the same space, has been shown to improve resource use efficiency and increase overall yields (**Figure** **4**).^[^ ^91^ ^]^ Legumes and cereals are commonly paired in intercropping systems because legumes have the unique ability to fix atmospheric nitrogen, which benefits nitrogen‐demanding crops like cereals. The deep mechanism here is that intercropping promotes complementary nutrient cycling, where legumes replenish soil nitrogen, reducing the need for synthetic fertilizers. Additionally, the spatial arrangement of different crops in intercropping systems can enhance light, water, and nutrient capture by utilizing different layers of the soil profile and canopy structure. This increased efficiency in resource use translates into higher overall yields, as both crops benefit from the shared environment without directly competing for the same resources.^[^ ^92^ ^]^

Agroforestry, which incorporates trees and shrubs into agricultural systems, also offers notable advantages. The deep mechanism of agroforestry lies in the interaction between trees and crops, where trees help improve soil fertility through the addition of organic matter via leaf litter and root biomass. Trees can fix atmospheric nitrogen, enriching the soil, which benefits subsequent crops, particularly those that require nitrogen for growth. Additionally, the root systems of trees and shrubs penetrate deeper into the soil compared to shallow‐rooted crops, allowing for better nutrient uptake and reduced competition for water and nutrients. This deeper root structure also helps in preventing soil erosion, particularly on sloped terrains, by binding the soil together and reducing surface runoff. Moreover, trees in agroforestry systems can create microclimates that protect crops from harsh environmental conditions, such as excessive sunlight or wind, further promoting biodiversity by supporting a variety of plant and animal species. These trees can serve as habitats for pollinators, beneficial insects, and wildlife, thus contributing to ecological stability and promoting overall farm resilience.^[^ ^93^ ^]^

Organic farming practices, which often involve diverse crop systems, align with sustainability goals by improving soil health and reducing dependence on synthetic inputs. The underlying mechanism of organic farming lies in the diversity of crops and the avoidance of synthetic fertilizers and pesticides. Diverse crop systems in organic farming enhance soil microbial activity by creating a variety of organic materials (from roots, leaves, and crop residues), which feed soil microorganisms, thereby improving soil structure and fertility. This increased microbial activity contributes to better nutrient cycling, which reduces the need for external fertilizer inputs and promotes long‐term soil health. Moreover, the practice of rotating crops and using cover crops in organic systems helps in maintaining a balance of soil nutrients and minimizing soil degradation. The absence of synthetic pesticides in organic farming also leads to the promotion of natural pest control, where beneficial insects thrive and contribute to pest management, enhancing biodiversity.^[^ ^94^ ^]^ Finally, organic systems, characterized by their crop diversity, lead to better soil quality and greater biodiversity. This is because biodiversity within the farming system supports a variety of ecological services, including pest control, pollination, and soil fertility maintenance. Diverse crop systems ensure that various ecological niches are filled, supporting a wide range of organisms from microbes to larger wildlife. These organisms contribute to a more balanced and resilient ecosystem, which helps the farm better withstand environmental shocks and reduces vulnerability to pest outbreaks and diseases.^[^ ^95^ ^]^

## 4 The Role of Crop Diversification in Enhancing Nutritional Security and Stabilizing Food Production

Crop diversification significantly boosts nutritional security by expanding dietary choices and enhancing nutrient intake (**Table** **2**). The deep mechanism behind this improvement lies in the ability of diversified cropping systems to produce a variety of nutrient‐dense foods, such as legumes, fruits, and vegetables, which supply a broader range of essential vitamins and minerals compared to monoculture systems.^[^ ^96^ ^]^ For instance, legumes like beans and lentils are rich in protein and iron, which address protein‐energy malnutrition and anemia, while fruits and vegetables contribute vital micronutrients like vitamin C, potassium, and dietary fiber.^[^ ^97^ ^]^ These nutrients play critical roles in bodily functions, including immune support, growth, and cognitive development. Moreover, the biochemical diversity in crops enhances nutrient bioavailability. For example, vitamin A in orange‐fleshed sweet potatoes or the zinc in certain legumes is more effectively absorbed when consumed as part of a diverse meal. Nutrient interaction mechanisms such as the synergistic effects of consuming vitamin C‐rich fruits with iron‐rich legumes increase iron absorption, directly combating deficiencies.^[^ ^98^ ^]^ In regions reliant on staple crops like rice or wheat, which often lack sufficient micronutrients such as zinc or vitamin A, crop diversification can address hidden hunger by incorporating crops naturally rich in these nutrients. For instance, adding bio‐fortified maize or iron‐rich millet to local diets can significantly enhance nutritional intake.^[^ ^99^ ^]^

**Table 2:** The Impact of Crop Diversification on Food Security.

Benefit Aspect of Crop Diversification	Statistical Evidence	Improvement in Food Security
Reduced Risk of Crop Failure	Diversifying crops can reduce the risk of yield loss by up to 30% during pest and disease outbreaks.^[^ ^110^ ^]^	Reducing crop failure risk ensures a more stable and reliable food supply, enhancing food availability.
Improved Nutritional Security	Crop diversification can increase dietary diversity scores by 15–20%.^[^ ^111^ ^]^	Increased dietary diversity improves nutrition, leading to better health outcomes and food security.
Local Food Systems	Increasing local crop diversity can reduce food imports by up to 40% and stabilize local food prices.^[^ ^112^ ^]^	Reducing reliance on food imports and stabilizing local food prices contribute to greater local food security.
Enhanced Soil Health and Fertility	Diversified cropping systems can increase soil fertility by up to 25% and reduce soil erosion.^[^ ^113^ ^]^	Healthier, more fertile soils improve crop yields and productivity, leading to greater food availability and stability.
Increased Farm Resilience to Climate Change	Crop diversification can enhance farm resilience, potentially increasing yields by up to 15% under climate change.^[^ ^35^ ^]^	Resilience to climate variability reduces the risk of crop failures, ensuring more stable food supplies and availability.
Improved Economic Stability for Farmers	Diversification can lead to a 10–20% increase in farm income stability.^[^ ^114^ ^]^	Economic stability for farmers allows for continued investment in productive practices, supporting a reliable food supply.
Support for Biodiversity Conservation	Diversified systems can support a 30–40% increase in agricultural biodiversity.^[^ ^115^ ^]^	Enhanced biodiversity supports ecosystem services, leading to stable crop yields and sustainable food production systems.
Enhanced Pollination Services	Crop diversification can improve pollinator activity by 20–30%.^[^ ^116^ ^]^	Improved pollination enhances crop yields and quality, directly contributing to food availability and diversity.
Reduced Soil Degradation	Diversification can reduce soil degradation by up to 25%.^[^ ^117^ ^]^	Preventing soil degradation maintains long‐term agricultural productivity, supporting consistent food production.

Additionally, diversified cropping systems improve dietary resilience. When one crop fails due to adverse weather or pests, others provide a safety net, ensuring a steady food supply rich in diverse nutrients. This adaptability not only improves food security but also ensures consistent nutrient availability across seasons. However, the extent of these nutritional benefits depends on local dietary habits, cultural preferences, and access to diverse produce in markets. For example, areas without adequate storage and transport infrastructure may face post‐harvest losses of perishable nutrient‐rich crops, such as fruits and vegetables, which undermines the benefits of diversification. Investing in cold storage and efficient logistics systems can mitigate these challenges and further enhance the nutritional impacts of crop diversification.^[^ ^100^ ^]^ Crop diversification plays a critical role in stabilizing food production and reducing the risks associated with monoculture systems. Diversified cropping systems distribute ecological and economic risks across multiple crops, ensuring greater resilience to environmental disruptions such as droughts, floods, or pest outbreaks. For example, drought‐resistant crops like millet can offset losses in water‐intensive crops like rice during dry spells, creating an ecological buffer against adverse conditions. This strategy acts as a form of insurance, ensuring that the failure of one crop does not jeopardize overall production.^[^ ^101^ ^]^ Diversified systems enhance yield stability by optimizing resource use and fostering ecological balance. Intercropping, where different crops are grown together creates microclimatic variations that improve soil moisture retention and reduce pest proliferation. For instance, legume‐cereal combinations not only stabilize yields but also enhance soil fertility through nitrogen fixation, benefiting the associated cereal crops. Such practices mitigate risks by spreading the burden across multiple crops, reducing the likelihood of total crop failure. In regions with highly variable climates, crop diversification proves especially beneficial.^[^ ^102^ ^]^ By cultivating a range of crops with varying tolerances to environmental stresses, farmers can ensure consistent production even under extreme conditions. Drought‐tolerant or flood‐resistant varieties, for example, sustain yields when unpredictable weather patterns disrupt conventional farming practices. Conversely, in areas with stable climates, the benefits of diversification may be less pronounced, as consistent environmental conditions diminish the need for adaptive strategies.^[^ ^103^ ^]^ The success of diversification depends significantly on the strategic selection of complementary crops. Combining deep‐rooted and shallow‐rooted species, for instance, optimizes water and nutrient use, reducing competition for resources and enhancing overall system resilience. However, poorly chosen crop combinations can lead to resource conflicts, diminishing the advantages of diversification. Properly managed systems balance ecological and agronomic needs, contributing to long‐term stability.^[^ ^95^ ^]^

Crop diversification also reduces pest and disease pressures, a significant vulnerability in monoculture systems. Diversified fields disrupt the lifecycle of pests and pathogens by creating less favorable environments for their proliferation.^[^ ^90^ ^]^ This approach minimizes the risk of widespread outbreaks, further stabilizing food production and reducing reliance on chemical pest control. While crop diversification offers significant benefits, its effectiveness depends on local conditions, crop selection, and market access. Farmers must carefully balance the advantages of increased stability with the management complexities and logistical challenges that diversified systems may entail. Nonetheless, by spreading risks, optimizing resource use, and enhancing ecological resilience, crop diversification emerges as a powerful tool for stabilizing food production and fostering sustainable agricultural systems.^[^ ^104^ ^]^

Crop diversification profoundly influences food supply chains and market access by introducing dynamic mechanisms that enhance resilience and efficiency at both local and global levels. Locally, diversified farming systems act as a buffer against food insecurity by increasing the availability of a broader spectrum of fresh produce. By cultivating a variety of crops, these systems minimize the dependency on singular external food sources, creating a self‐reliant food ecosystem. This mechanism ensures that communities have a stable supply of essential nutrients, even during disruptions in external supply chains.^[^ ^105^ ^]^ Furthermore, diversified systems strengthen local food systems by fostering self‐sufficiency and reducing the vulnerability to market or logistical disturbances. This is achieved through increased production of diverse crops tailored to local demands, thereby mitigating the risk of shortages or price surges. The reduced reliance on monocultures prevents total system collapse in the face of crop‐specific failures, ensuring consistent supply. Studies by Hendrickson^[^ ^106^ ^]^ and Kahiluoto^[^ ^107^ ^]^ highlight how these systems decrease the likelihood of supply disruptions, promoting steady access to food resources. On a larger scale, crop diversification builds market resilience by ensuring diverse products flow through supply chains, reducing the impacts of global shocks such as pandemics, extreme weather events, or geopolitical crises. These mechanisms collectively reinforce food security by creating more stable and adaptable markets. Lioutas and Charatsari;^[^ ^108^ ^]^ Gonzalez^[^ ^109^ ^]^ further demonstrate that diversified farming systems enhance the capacity of local markets to withstand external shocks, protecting community access to food during crises.

## 5 Limitations, Challenges, and Solutions in Implementing Crop Diversification

Farmers and agricultural stakeholders encounter numerous challenges and limitations when adopting crop diversification. These issues stem from gaps in knowledge and skills, market and infrastructure limitations, policy and institutional barriers, and environmental constraints, all of which affect the feasibility and success of diversification efforts. One significant limitation is the potential trade‐off between diversification and economic specialization. While diversification improves resilience and reduces risk, it may lower short‐term profitability for farmers who are accustomed to high‐yield monoculture crops.^[^ ^118^ ^]^ Additionally, labor requirements can increase significantly with diversified systems, as managing multiple crops often involves more intensive planning, maintenance, and harvesting. This can pose a particular challenge for smallholder farmers with limited access to labor resources. Another economic hurdle is the high initial investment required to shift to diversified systems, which often necessitates substantial financial inputs for seeds, tools, and other resources. This can discourage farmers, particularly those with limited financial capacity, from adopting diversified practices.^[^ ^119^ ^]^

Knowledge gaps also represent a critical obstacle. Successful crop diversification demands a deep understanding of crop selection, interactions, and best management practices. However, many farmers lack the technical expertise to make informed decisions, resulting in suboptimal diversification efforts and potential financial losses. To bridge this gap, comprehensive training and extension services are crucial. These programs should focus on practical management techniques, risk assessment, and sustainable practices.^[^ ^120^ ^]^ Evidence suggests that well‐structured training initiatives can enhance farmers’ capabilities, boosting both productivity and system sustainability. Market access is another challenge. Farmers often face difficulties in finding buyers for less common or newly introduced crops. Market unpredictability and price volatility can deter diversification efforts, as farmers struggle to recover investments in diversified systems.^[^ ^120^ ^]^ Inadequate infrastructure for storage, transportation, and marketing further exacerbates these challenges. Poor infrastructure leads to high post‐harvest losses and increased operational costs, making it harder for farmers to compete in the marketplace. To address these issues, investments in infrastructure are critical. This includes developing storage facilities, improving transportation networks, and enhancing market linkages to support diversified farming systems.

Policy and institutional limitations also play a significant role. The absence of targeted policies, subsidies, and research funding can slow the adoption of crop diversification. Many regions lack supportive frameworks that incentivize diversification or provide financial and technical assistance to farmers.^[^ ^121^ ^]^ Institutional barriers, such as limited access to credit, inputs, and technical support, further impede the adoption of diverse cropping systems. Addressing these barriers requires proactive policies that offer financial incentives, technical training, and research support. Strengthening institutional support can enhance the scalability and sustainability of crop diversification practices. Environmental and climatic constraints also limit the effectiveness of crop diversification. Variations in temperature, precipitation, and extreme weather events caused by climate change influence the viability of diversified systems.^[^ ^122^ ^]^ In addition, local environmental factors such as soil type, water availability, and pest pressures may restrict the selection of suitable crops for diversification. Developing strategies that account for these limitations, such as selecting climate‐resilient crop varieties and adapting planting schedules, is essential for mitigating risks.^[^ ^123^ ^]^ Despite its many advantages, crop diversification is not a one‐size‐fits‐all solution. Economic, social, and ecological constraints must be carefully considered and addressed through targeted interventions. By providing farmers with the necessary knowledge, resources, and market access, as well as by designing supportive policies and infrastructure, the limitations and challenges associated with crop diversification can be effectively managed, paving the way for more sustainable agricultural systems.^[^ ^124^ ^]^

## 6 Global Insights into Crop Diversification: Successes, Challenges, and Lessons Learned

Crop diversification has yielded varying degrees of success across different regions, offering valuable insights into effective practices and potential challenges (**Table** **3**). In Southeast Asia, integrated rice‐fish farming stands out as a successful diversification model.^[^ ^125^ ^]^ This system combines rice cultivation with fish farming, utilizing their ecological interactions to boost productivity. The fish help control pests and enrich the soil with essential nutrients, enhancing rice yields and promoting biodiversity. This model improves water management and ecological balance, showing its effectiveness in increasing overall productivity.^[^ ^126^ ^]^ In Latin America, the integration of shade trees into coffee plantations has proven beneficial. These trees provide shade to protect coffee plants from excessive heat, enhance soil health, reduce erosion, and support wildlife. Consequently, shade trees improve both coffee yields and quality, demonstrating how complementary crops can enhance agricultural productivity and ecosystem health.^[^ ^127^ ^]^

**Table 3:** Crop Diversification Models in Different Regions a long with their Key Benefits.

Region	Diversification Model	Key Benefits	References
Southeast Asia	Integrated rice‐fish farming	Enhanced productivity, improved water management, increased biodiversity	[125, 126]
Latin America	Shade trees in coffee farms	Improved soil health, reduced erosion, enhanced coffee yields and quality	[129]
Sub‐Saharan Africa	Agroforestry (trees with maize and beans)	Increased soil fertility, better moisture retention, higher crop yields, additional income	[128]
India	Diversified vegetable farming alongside staples	Reduced economic risks, improved soil health, steady income year‐round	[130]
East Asia	Aquaponics (fish farming with hydroponics)	Enhanced resource efficiency, improved sustainability	[131]
Middle East	Under‐cropping vegetables beneath date palms	Maximized land use, diversified income streams, improved soil quality, reduced water usage	[132]
Australia	Cover cropping and rotation systems	Enhanced soil health, effective nutrient management, increased crop yields, pest resilience	[133]

Sub‐Saharan Africa has witnessed notable success with agroforestry systems that integrate trees with crops like maize, beans, and other staples, significantly improving soil fertility, moisture retention, and overall agricultural productivity. For instance, in Kenya, smallholder farmers have successfully incorporated *Grevillea robusta* trees into their maize and bean fields, enhancing soil fertility through nitrogen fixation and reducing erosion. Similarly, in Ethiopia, the integration of *Faidherbia albida* trees with maize farming in regions like Tigray and Amhara has led to a significant increase in crop yields, as the trees improve soil fertility and moisture retention. In Tanzania, *Faidherbia albida* has also been used successfully, with trees shedding leaves during the growing season, allowing crops to benefit from sunlight and nutrients while enriching the soil. Additionally, in Ethiopia's Southern Nations, Nationalities, and Peoples' Region (SNNPR), *Sesbania sesban* trees have been integrated with crops to improve soil health, provide green manure, and offer multiple income sources through fuelwood and fodder.^[^ ^128^ ^]^

In India, growing diverse vegetables alongside staple crops like wheat and rice reduces economic risks and enhances soil health, providing a steady income throughout the year.^[^ ^134^ ^]^ In East Asia, Taiwan's innovative aquaponics system, which combines fish farming with hydroponics, serves as an example of a novel horticultural approach. In this system, ammonia waste is produced by fish and converted into nitrates by beneficial bacteria in plant grow beds. Vegetables use this nitrate for growth, while the water is cleaned and recirculated back to the fish tanks. This process enhances resource efficiency and sustainability by maintaining a clean and balanced environment for both fish and plants.^[^ ^135^ ^]^ In the Middle East, particularly in Saudi Arabia and the UAE, cultivating vegetables beneath date palms has optimized land use and diversified income streams. This practice improves soil quality and reduces water usage, demonstrating how strategic crop placement can maximize resource efficiency.^[^ ^136^ ^]^ Australia's use of cover cropping and rotation systems further illustrates successful diversification. Incorporating legumes and other cover crops enhances soil health, manages nutrients effectively, and boosts crop yields while increasing resilience against pests and diseases.^[^ ^137^ ^]^ However, not all diversification efforts have been successful. Challenges such as inadequate market demand, insufficient knowledge, and poor planning can hinder success. For example, the cultivation of Jatropha for biofuel in Africa faltered due to overestimated market demand and poor yields, resulting in minimal profit for farmers.^[^ ^138^ ^]^ In Bolivia, the rapid expansion of quinoa cultivation led to market saturation and price drops, adversely affecting local farmers who had neglected other crops.^[^ ^139^ ^]^ In India, attempts to diversify with papaya struggled as farmers faced severe pest and disease issues and volatile market prices due to insufficient training.^[^ ^140^ ^]^ Additionally, the expansion of rubber plantations in Southeast Asia exposed smallholders to global price fluctuations and left them with no alternative income during the crop's lengthy maturation period.^[^ ^141^ ^]^ These examples underscore the need for thorough market research, feasibility studies, and access to training and resources to overcome obstacles and achieve positive outcomes.

## 7 Conclusion 

In conclusion, navigating the contemporary challenges in agriculture calls for a strategic shift towards crop diversification. This approach presents a viable solution to mitigate the risks posed by climate change, pest pressures, and economic volatility. By moving away from monoculture and embracing diversified cropping systems, farmers can significantly enhance agricultural resilience, promote sustainability, and strengthen food security. The principles underpinning crop diversification—ecological resilience, risk management, and resource optimization—reveal its myriad benefits. These include improved soil health, effective pest management, and increased economic stability. Despite these clear advantages, the path to implementing crop diversification is fraught with challenges, such as gaps in knowledge, market access constraints, and the need for supportive policies. Successful examples from various regions demonstrate that diversified cropping systems can markedly boost productivity, ecological balance, and income stability. Overcoming these challenges through targeted investments, comprehensive support mechanisms, and robust policy frameworks will be essential for the widespread adoption of crop diversification. Such efforts will contribute to more resilient and sustainable agricultural practices, ensuring a more secure and productive future for the global food system.

## 8 Future Directions, Policy Options, and Research Priorities

### 8.1 Future Directions and Policy Options

Effective promotion of crop diversification requires robust policy support. A critical step is enhancing rural infrastructure—such as transportation networks, electrification, and modern storage facilities—to attract private investment in agriculture. For example, Rwanda's investment in rural roads has significantly improved market access for smallholders, leading to increased private investment. Coupling infrastructure improvements with predictable trade policies can reduce market risks and make agriculture more appealing to investors. Another key strategy involves rebalancing public sector involvement to support a wider range of crops while reducing direct market control. In India, reducing the monopoly of certain government market boards has encouraged the cultivation of alternative crops. Additionally, revising input subsidy schemes that favor staple crops like maize is essential to prevent monoculture. Diversifying these subsidies to include various seeds would motivate smallholder farmers to adopt more sustainable and profitable cropping options. Revitalizing land policies is also crucial. Transparent policies that prioritize local farmers can address speculative land acquisitions. Ethiopia's land certification program, which has secured land tenure for millions of smallholders, is a prime example of fostering investment in diverse crops. Strengthening land rights will enable smallholders to optimize land use, promoting long‐term investment in diversified agriculture. To further support crop diversification, financial incentives and subsidies can offset initial adoption costs. Increased investment in research is necessary to optimize diversification practices and develop innovative technologies. Extension services and technical support are critical in helping farmers successfully implement and manage diversified cropping systems.

### 8.2 Future Research Focus

While crop diversification is known to enhance sustainable agriculture, several research gaps remain. Long‐term studies are needed to evaluate the impacts of diverse cropping systems on soil health and ecosystem stability. Detailed economic analyses should assess the financial implications of initial investments versus long‐term benefits, particularly for smallholder farmers. Research should also explore how diversification strategies can be adapted to specific climatic conditions, such as extreme weather events, to manage climate risks effectively. Additionally, the effectiveness of policy interventions—such as subsidies and technical assistance—in promoting crop diversification needs further investigation. Understanding how cultural factors and farmers' perceptions influence the adoption of diversified cropping systems is another key area of research. Studies should focus on improving market access, developing efficient supply chains, and exploring consumer preferences for diverse crops. Moreover, the impact of educational programs and extension services on farmers' ability to manage diversified systems warrants further investigation. Finally, precise methods are needed to measure the ecosystem services provided by diversified systems, such as pollination and pest control, and to explore how technological innovations can enhance their management. Addressing these research gaps will deepen the understanding of crop diversification's benefits and limitations, leading to more effective strategies for sustainable agriculture and food security.

## Conflict of Interest

The authors declare no conflict of interest.
